# *In vitro *antiproliferative activity of partially purified *Trigona laeviceps *propolis from Thailand on human cancer cell lines

**DOI:** 10.1186/1472-6882-11-37

**Published:** 2011-05-06

**Authors:** Supawadee Umthong, Preecha Phuwapraisirisan, Songchan Puthong, Chanpen Chanchao

**Affiliations:** 1 Department of Biology, Faculty of Science, Chulalongkorn University, 254 Phayathai Road, Bangkok 10330, Thailand; 2 Department of Chemistry, Faculty of Science, Chulalongkorn University, 254 Phayathai Road, Bangkok 10330, Thailand; 3 Institute of Biotechnology and Genetic Engineering, Chulalongkorn University, 254 Phayathai Road, Bangkok 10330, Thailand

## Abstract

**Background:**

Cancers are some of the leading causes of human deaths worldwide and their relative importance continues to increase. Since an increasing proportion of cancer patients are acquiring resistance to traditional chemotherapeutic agents, it is necessary to search for new compounds that provide suitable specific antiproliferative affects that can be developed as anticancer agents. Propolis from the stingless bee, *Trigona laeviceps*, is one potential interesting source that is widely available and cultivatable (as bee hives) in Thailand.

**Methods:**

Propolis (90 g) was initially extracted by 95% (v/v) ethanol and then solvent partitioned by sequential extractions of the crude ethanolic extract with 40% (v/v) MeOH, CH_2_Cl_2 _and hexane. After solvent removal by evaporation, each extract was solvated in DMSO and assayed for antiproliferative activity against five cancer (Chago, KATO-III, SW620, BT474 and Hep-G2) and two normal (HS27 fibroblast and CH-liver) cell lines using the MTT assay. The cell viability (%) and IC_50 _values were calculated.

**Results:**

The hexane extract provided the highest *in vitro *antiproliferative activity against the five tested cancer cell lines and the lowest cytotoxicity against the two normal cell lines. Further fractionation of the hexane fraction by quick column chromatography using eight solvents of increasing polarity for elution revealed the two fractions eluted with 30% and 100% (v/v) CH_2_Cl_2 _in hexane (30DCM and 100DCM, respectively) had a higher anti-proliferative activity. Further fractionation by size exclusion chromatography lead to four fractions for each of 30DCM and 100DCM, with the highest antiproliferative activity on cancer but not normal cell lines being observed in fraction# 3 of 30DCM (IC_50 _value of 4.09 - 14.7 μg/ml).

**Conclusions:**

*T. laeviceps *propolis was found to contain compound(s) with antiproliferative activity *in vitro *on cancer but not normal cell lines in tissue culture. The more enriched propolis fractions typically revealed a higher antiproliferative activity (lower IC_50 _value). Overall, propolis from Thailand may have the potential to serve as a template for future anticancer-drug development.

## Background

Cancers are some of the major fatal diseases to humans. Chemotherapy is one of the most widely used approaches for the treatment of many cancers, but the long-term use of chemotherapy can lead to drug resistance via several different mechanisms, such as gene mutation, DNA methylation and histone modification. These resistance mechanisms have been reported to play important roles in the resistance of cancers to chemotherapeutic agents [1]. Thus, patients are gradually developing resistance to widely used and standard chemotherapeutic agents, such as 5-fluorouracil, taxol, doxorubicin, cisplatin, campothecin, paclitaxel and topotecan [2]. Due to this resistance to cancer drugs, it is important to find new anticancer agents in order that they can be developed into novel anticancer drugs that can circumvent the existing resistance mechanisms. Herbs and other natural plant products have become interesting sources for this purpose, but animal modified or selected plant products have been largely overlooked.

Propolis, one of the economic natural products from bees, is an interesting source for several bioactivities, such as antimicrobial as well as anti-cancer. Although an animal product it is largely plant based in its chemical origins. It is a sticky resin and varies in colour, including brown, green and red amongst others, based upon the plant exudates that the bees have selectively collected from flower buds, leaf buds and tree barks. These plant resins are mixed with waxes and other bee excretions, including enzymes [3], to form the final propolis product. Although used as a sealing wax for filling cracks and repairing combs, and in some cases embalming wax, the principal use of propolis in the beehive is as a protective barrier against their enemies [4]. As such it has broad antimicrobial activities and has been extensively used in the traditional medicine [5]. In Europe, propolis was accepted as an official drug due to its antibacterial activity during the last 400 years [6]. Furthermore, propolis has been long used as a dietary supplement for disease prevention [7], since it can provide antimicrobial activity, including antiviral activity [8], anti-inflammatory [9], immunomodulatory [10], antitumor [11] and antioxidant effects [12].

The chemical composition of each type of propolis and its associated bioactivities mainly depend on the macro- and micro-geographical regions, due to the differences in the plant resin compositions or available plant species [8,13], and on the bee species, due to the different preference for food and resin plants and foraging distances between bee species [14]. For example, the pollen in the propolis from *Apis mellifera *in the Preveza region of Northwest Greece was mainly from Pinaceae [14] whilst in the propolis in Brazil it was mainly from two poplar trees, *Hyptis di *V*aricata *and *Baccharis dracunculifolia *[15,16], suggesting quiet different sources of plant resins and volatiles for the propolis production. In addition, the propolis from *A. mellifera *in Brazil, Chili and Myanmar presented a different *in vitro *cytotoxicity activity against the PANC-1 human pancreatic cancer cells in a nutrient-deprived medium [16-18].

Although propolis is typically a complex mixture of diverse compounds from plant resins, volatiles, pollen and animal enzymes, etc, numbering over 300 characterized components, the reported bioactivities associated with propolis have been found in both the crude and the purified extracts [12,19], suggesting that they may be associated with single compounds rather than complex interactions between compounds, and so amenable to purification. To date, most of the active compounds from purified extracts have been found to be phenolics and polyphenolics [20]. Propolin A and propolin B, which belong to the prenylflavanone group, were the main chemical components that could be isolated using Nuclear Magnetic Resonance (NMR) from Taiwanese propolis [21]. Both components displayed an *in vitro *antiproliferative effect on human melanoma, C6 glioma and HL-60 cell lines in tissue culture.

Caffeic acid phenethyl ester (CAPE), the main component from *A. mellifera *propolis in Chili, along with a considerable number of flavonoid compounds, such as *p*-cumaric acid and ferulic acid, were purified and found to display an *in vitro *antiproliferative effect on the KB, DU-145 and Caco-2 cell lines in tissue culture [12].

Given that the main active components found to date are plant derived flavonoids, it would seem likely to be better to preserve the polyphenolic fraction of propolis after purification.

Propolis from the stingless bee *Trigona laeviceps *Smith (Hymenoptera: Apoidae) was used in this research since these bees can be commercially cultivated in a sustainable and potentially ecologically friendly manner in artificial hives (with the potential add on value of certain crop plant pollination), and can provide a lot of propolis per hive. In addition, the bees are widely distributed throughout Thailand. Although Umthong *et al. *reported that a high cytotoxicity on the SW620 colon cancer cell line was obtained from the crude extract of propolis harvested from *T. laeviceps *in Thailand [22], it is not recommended to use or consume propolis in the form of a crude extract because it may still contain various adverse bioactivities. In this research, we attempted to purify the ethanol crude extract of *T. laeviceps *propolis from Samut Songkram, central Thailand, using a bioassay-guided isolation procedure. Solvent partitioning of the propolis, based upon solvent polarity, was performed and human cell lines derived from five different types of tissue cancers were used to screen for any *in vitro *antiproliferative affect in comparison to the two normal cell lines.

## Methods

### Sample collection

Propolis of *T. laeviceps *was collected from an apiary in the Samut Songkram province, central Thailand, and was kept in the dark at 4°C until use.

### Ethanol crude extraction

The method of propolis extraction followed that reported by Najafi *et al*. [23]. Briefly, propolis (90 g) was cut into small pieces and was then extracted by 95% (v/v) ethanol (400 ml) at 15°C with shaking at 100 rpm for 20 h. The suspension was then clarified of residual propolis solid by centrifugation at 7,000 rpm for 15 min at 20°C. The supernatant was harvested and kept whilst the pellet was re-extracted and then clarified as above except using 100 ml and not 400 ml of 95% (v/v) ethanol. The two ethanolic extracts (supernatants) were pooled together and evaporated in a rotary evaporator (40°C). The obtained residue (crude ethanolic extract) was weighed and stored at -20°C at dark.

### Partial purification of the crude ethanolic extract of propolis by solvent partitioning with different polarity solvents

The ethanolic extract of the propolis was dissolved in 80% (v/v) methanol until it was not sticky and then an equal volume of hexane was added, stirred (15 mins) and then allowed to phase separate in a separating funnel. The upper hexane phase containing the non-polar compounds was harvested and kept whilst the 80% (v/v) methanol phase containing polar compounds was extracted three more times with hexane in the same manner. The four hexane extracts were pooled, the solvent evaporated in a rotary evaporator (40°C), and the sticky liquid residue weighed to give the yield of the hexane extract. The 80% (v/v) methanol phase was then mixed with an equal volume of H_2_O to increase the partition coefficient (solubility) of polar compounds, and then extracted four times with an equal volume of CH_2_Cl_2 _as per hexane above, except that following phase separation the CH_2_Cl_2 _phase containing the less polar compounds was the lower phase. The pooled CH_2_Cl_2 _phases and the residual 40% (v/v) methanol phase were separately evaporated to remove the respective solvent in a rotary evaporator (40°C), and the residues were each weighed to give the yield of the CH_2_Cl_2 _and methanolic extracts, respectively.

### Chromatography

#### Quick column chromatography

Silica gel was packed into a glass funnel (50% of internal volume) connected to a vacuum pump. The selected fractions (see Bioassay guided selection below) were mixed with CH_2_Cl_2 _until they were not sticky and were then mixed with silica gel, evaporated to dryness and loaded on top of the silica gel containing column. The column was then eluted by various solvents (2,000 ml each) of different polarities starting from the least polar solvent and increasing, that is from 0:1, 1:9, 3:7, 1:1, 7:3 and 1:0 (v/v) of CH_2_Cl_2_: hexane and then followed by 5% and 10% (v/v) MeOH in CH_2_Cl_2_, respectively. A reduced pressure was used in order to enable the flow rate of the solvent through the column to be obtained, and fractions were collected. Each fraction was solvent evaporated in a rotary evaporator (40°C), and the residue weighed before being dissolved in DMSO to the required concentrations to assay for antiproliferative activity as detailed below.

#### Size exclusion chromatography

Further partial purification of each selected fraction was performed by size exclusion chromatography using a Sephadex LH-20 column (10 ml internal volume). Each selected fraction was dissolved in a 1:1 (v/v) ratio of MeOH: CH_2_Cl_2 _until it was not sticky and was loaded on top of the column. The column was then eluted with 10 ml of a 1:1 (v/v) ratio of MeOH: CH_2_Cl_2_, collecting 2 ml fractions. The four fractions obtained were then solvent evaporated by a rotary evaporator (40°C), the residue weighed and then dissolved in DMSO to the required concentrations ready to assay for any selective antiproliferation activity as detailed below.

#### Thin layer chromatography (TLC)

One-dimensional thin layer chromatography (TLC) was used in order to group the obtained components and to determine the purity of fractions. A silica gel plate (10 cm in height) was used as the stationary phase, and the respective extracts or fractions were spotted at the starting line at 0.5 - 1 cm intervals. The mobile phase solvents used (one per TLC plate) were 1:0, 3:1 and 1:1 (v/v) ratio of CH_2_Cl_2_: hexane. When the mobile phase had almost reached the top of the plate, the samples were visualized under U.V. light (254 nm). Alternatively, the gel plate was sprayed by a 5% (v/v) H_2_SO_4_/0.03% (w/v) α-naphtal methanolic solution, dried in an oven or hot plate and visualized under U.V. light (350 nm).

### Bioassay-guided isolation

Each of the three crude solvent partitioned extracts (hexane, dichloromethane and methanolic) of propolis, obtained as detailed above, were evaluated for their *in vitro *antiproliferative activity on five selected cancer and two normal cell lines in tissue culture using the MTT assay as detailed below. The extract which provided the best selective antiproliferative activity, that is the highest activity on the cancer cell lines but not on the normal cell lines, as determined by comparison of the IC_50 _values, was selected for further partial purification by quick column silica chromatography. In the same way, each fraction obtained from the quick column chromatography was likewise assayed for selective antiproliferative activity on the cell lines and this was used to select fractions for further size exclusion chromatography (see above).

### Cancer cell lines

The five selected cancer cell lines for screening for the *in vitro *antiproliferative bioactivity were derived from colon (SW620), breast (BT474), hepatic (Hep-G2), lung (Chago), and stomach (Kato-III) tissue cancers. The two normal cell lines used were of liver (CH-liver) and fibroblast (HS-27) origins and were used as comparative controls to check for selective specificity towards cancer cells rather than all dividing cells. All cell lines were cultured in RPMI medium containing 5% (v/v) fetal calf serum (complete medium) at 37°C in a humidified air atmosphere containing 5% (v/v) CO_2_.

### MTT assay for proliferation

Cultured cells (5,000 cells) in 200 μl complete media were transferred into each well of a flat 96 well plate and then incubated at 37°C in a humidified air atmosphere enriched with 5% (v/v) CO_2 _for 24 h in order to let the cells attach to the bottom of each well. The cultured cells were then treated with the tested propolis extract (triplicate wells per condition) by the addition of 2 μl of serial dilutions of the propolis extract dissolved in DMSO to give a final concentration of 100, 50, 25, 12.5, 6.25 and 3.125 μg/ml. In addition, 2 μl of DMSO alone was added to another set of cells as the solvent control. The cells were then cultured as above for another 48 h prior to the addition of 10 μl of a 5 mg/ml solution of 3-(4, 5-dimethylthiazol-2-yl)-2, 5-diphenyltetrazolium bromide (MTT)into each well. The incubation was continued for another 4 h before the media was removed. A mixture of DMSO (150 μl) and glycine (25 μl) was added to each well and mixed to ensure cell lysis and dissolving of the formasan crystals, before the absorbance at 540 nm was measured. Three replications of each experiment were performed and the half maximal inhibitory concentration (IC_50_) of each extract was calculated as detailed below.

### Half maximal inhibitory concentration (IC_50_)

The obtained absorbance at 540 nm was used to determine the percentage of cell survival assuming that 100% survival was obtained from the solvent only control and that no differences in metabolic activity existed between surviving cells under the different conditions. Under these assumptions, the percentage survival of the treated cancer and normal cultured cells was calculated according to the formula below:

The mean (± 1 standard deviation (SD)) cell survival (%) was plotted against the corresponding propolis extract concentration and the best fit line was used to derive the estimated IC_50 _value from the concentration that could provide a 50% cell survival.

## Results

### Antiproliferation of ethanol crude extract

From 90 g of *T. laeviceps *propolis a yield of 18 g (20%) of crude ethanol extract was obtained as a sticky brownish to dark brown resin with a distinctive smell. When assayed *in vitro *on the five cancer and two normal cell lines, using the MTT assay, the IC_50 _(μg/ml) values for the five cancer cell lines ranged from 1.9-fold lower (SW620 and BT474) to only 1.04-fold lower (Hep-G2) than that for the control HS27 cell line (Table 1). However, the CH-Liver control cell line was some 1.3-fold more sensitive than the HS27 cell line and so only three of the cancer cell lines showed reduced IC_50 _(μg/ml) values compared to the control CH-liver cell line. Over all, it indicates that the crude ethanolic extract of *T. laeviceps *propolis contained some antiproliferative activity, but with the overlapping variation within the cancer and normal cell lines the existence of any selective specificity for cancer cells was less clear.

**Table 1:** Preferential cytotoxicity (IC_50 _value in μg/ml) of ethanol crude extract on five cancer cell lines and two normal cells

Cancer cell lines				Normal cells	
**BT474**	**Chago**	**SW620**	**Hep-G2**	**KATO-III HS27**	**CH-liver**

20.40	31.67	19.88	36.19	22.98 37.85	29.14

### Active fractions after solvent partitioning of the crude ethanolic propolis extract

**Figure 1:**
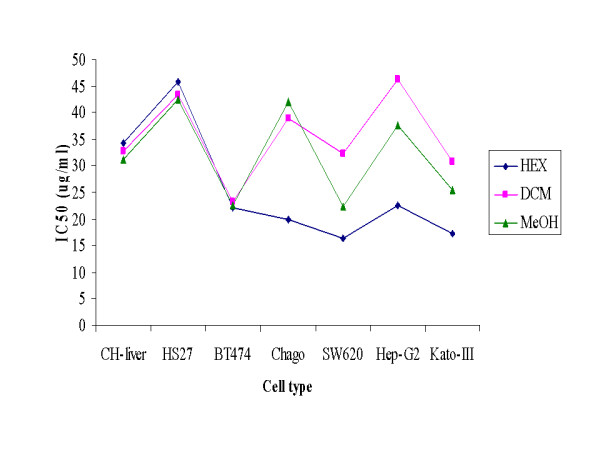
**Average *in vitro *IC_50 _values (μg/ml) of the three different solvent partitioned fractions (see Table 2) on the five cancer and two normal cell lines in tissue culture, as determined by the MTT assay**. Hex = hexane extract, DCM = CH_2_Cl_2 _extract and MeOH = methanol extract. Data came from the mean ± 1 SD of percentage of cell viability which was derived from three replicates.

After resolvation of the crude ethanolic extract in 80% (v/v) methanol, sequential solvent partitioning with three different polarity solvents was used to further fractionate the propolis extracts. For each of the three solvent extractions the new fractions obtained gave different appearances (Table 2), but the CH_2_Cl_2 _extract showed no significant antiproliferative bioactivity on four of the five cancer cell lines tested (Figure 1), whilst the methanol extract was only significantly effective against two of the five cancer cell lines. In contrast, the hexane extract, and thus the low polarity compounds, were the most affective, showing a strong antiproliferative affect on all five cancer cell lines above that seen on the two control cell lines (Figure 1).

**Table 2:** The obtained fractions of ethanol crude extract by partition

Solvent	Level of polarity	Characters of separated compounds
Hexane	Low	Light brown and sticky liquid
CH_2_Cl_2_	Medium	Dark brown and sticky solid
40% (v/v) MeOH	High	Very dark brown to black solid

### Further enrichment of the antiproliferation active compounds from the hexane-partitioned fraction by quick column chromatography

**Figure 2:**
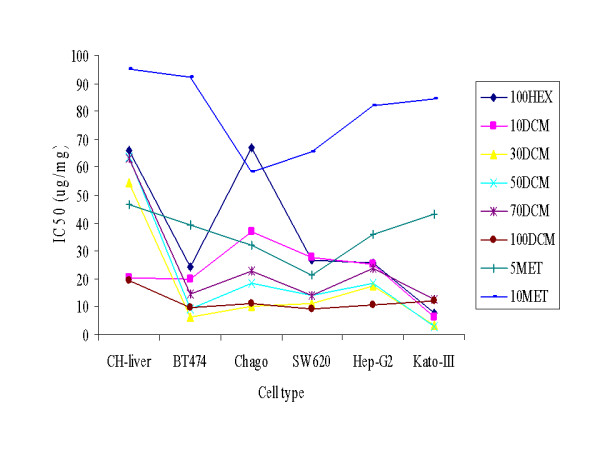
**Average IC_50 _(μg/ml) values of the eight fractions, obtained after quick column chromatography, on the five cancer and two normal cell lines**. 100HEX, 10DCM, 30DCM, 50DCM, 70DCM, 100DCM, 5MET and 10MET stand for the fractions eluted in 0:1, 1:9, 3:7, 1:1, 7:3 and 1:0 (v/v) ratios of CH_2_Cl_2_: hexane, and 1:19 and 1:9 (v/v) ratios of MeOH: CH_2_Cl_2_, respectively (see table 3). Data came from the mean ± 1 SD of percentage of cell viability which was derived from three replicates.

Given that the hexane partitioned fraction showed the greatest antiproliferative effect on the five tested cancer cell lines (Figure 1), it was selected for further enrichment by quick column chromatography. The eight fractions obtained from the different elution solvent mixtures revealed seven different appearances (70DCM and 100DCM were similar) (Table 3), but the highest polarity fraction, obtained from elution with 1:9 (v/v) ratio of methanol: CH_2_Cl_2 _revealed essentially no antiproliferative activity (Figure 2). In contrast, based on the IC_50 _(μg/ml) values, the fraction obtained from the 1:9 (v/v) ratio of CH_2_Cl_2_: hexane revealed a general antiproliferative effect and was not specific to the cancer cell lines. The pure hexane eluted fraction revealed a low cytotoxicity to the control CH-liver cells and a high cytotoxicity on four of the cancer cell lines, the exception being the Chago cell line (66.9 μg/ml compared to 66.2 μg/ml for the control CH-liver cell line). In contrast, the fractions eluted from the 3:9, 1:1 and 7:3 (v/v) ratios of CH_2_Cl_2_: hexane revealed a significant antiproliferation activity on all five cancer cell lines but only a slight affect on the control CH-liver cell line. From these three fractions that which eluted from the 3:7 (v/v) ratio of CH_2_Cl_2_: hexane (30DCM) was selected for further enrichment by size exclusion chromatography. In addition, the fraction that eluted in pure CH_2_Cl_2 _(100DCM) was selected for further enrichment. Although the 100DCM extract displayed a high antiproliferation activity on the control normal cell lines, it displayed a very high activity against all five cancer cell lines, and so was selected in case the two activities could be segregated by further enrichment.

**Table 3:** Eight compounds purified by quick column chromatography

Purified compounds	Characteristics
100HEX	yellow and green, sticky liquid
10DCM	transparent, light yellow and brown, sticky liquid
30DCM	transparent, light brown and sticky solid
50DCM	transparent, like 30DCM but lightly darker
70DCM	transparent, dark brown and sticky solid
100DCM	transparent, dark brown and sticky solid
5MET	brown and black solid
10MET	light yellow solid

### Further enrichment of the active compounds by size exclusion chromatography

As mentioned above, fractions 30DCM and 100DCM were the most active in the MTT based *in vitro *antiproliferation of cancer cell lines, and so were selected for size exclusion chromatography. For both 30DCM and 100DCM, after size exclusion chromatography, four positive fractions (F1 - F4) were obtained with varying appearances (Table 4). Comparing the color of these fractions after size exclusion chromatography to the color of crude extract in table 2, a lighter color was evident after size fractionation.

**Table 4:** Characters of isolated active fractions after size exclusion chromatography

Fraction	Characters
30DCM-F1	Yellow solid
30DCM-F2	Yellowish brown solid
30DCM-F3	Brown solid
30DCM-F4	Yellow solid
100DCM-F1	Reddish brown solid
100DCM-F2	Brown solid
100DCM-F3	Yellowish brown solid
100DCM-F4	Brown solid

These eight fractions (Table 4) revealed different antiproliferation activities on the five cancer cell lines as well as the control cell lines (Table 5). Fractions 1 and 4 from the 30DCM extract revealed no detectable activity on all tested cell lines, whilst fractions 1 and 2 from the 100DCM fraction showed only a weak activity against one or all, respectively, of the five cancer cell lines. Fractions 3 and 4 from the 100DCM extract were among the three most active fractions, but also showed a strong inhibition of the control cell lines. Thus, the antiproliferative affect of the 100DCM fraction on the cancer and normal cell lines was not segregated. In contrast, fractions 2 and 3 from the 30DCM extract showed a moderately and very strong antiproliferative affect on all five cancer cell lines but not the control cell lines (Table 5).

**Table 5:** Preferential cytotoxicity (IC_50 _value in μg/ml) of positive fractions after size exclusion chromatography on five cancer cell lines and two normal cells

Fraction	Cancer cell lines					Normal cells
	**BT474**	**Chago**	**SW620**	**Hep-G2**	**Kato-III**	**CH-liver**

30DCM-F1	>100	>100	>100	>100	>100	>100
30DCM-F2	18.70	48.64	27.84	13.5	18.07	57.77
30DCM-F3	9.50	14.67	9.20	10.93	4.09	80.15
30DCM-F4	>100	>100	>100	>100	>100	>100
100DCM-F1	73.70	>100	52.72	62.53	58.88	57.73
100DCM-F2	70.91	>100	>100	>100	67.85	>100
100DCM-F3	7.64	16.7	14.28	15.98	7.55	19.35
100DCM-F4	9.34	11.37	7.98	9.64	8.31	9.93

## Discussion

In this research, initial purification steps were performed on the propolis in order to enrich for the selective antiproliferative bioactive fraction, using a cell line antiproliferation assay as a guide. Ethanol was used as the extraction solvent to provide a crude propolis extract as discussed in Orsolic *et al*. [24]. In addition, Sawaya *et al*. reported that the soluble compounds could be easily released from the sticky part of propolis by a high percentage of alcohol [25]. Considering the crude ethanol extract of *T. laeviceps *propolis, not only was a cytotoxic affect on the five selected cancer cell lines seen but also on the two control normal cell types. The IC_50 _(μg/ml) between the inhibition of cancer and control cell line proliferation were close (Table 1), preventing useful anti-cancer application. A similar result has been observed in the crude aqueous extract of propolis from *A. mellifera *in Iran, where although it could inhibit the *in vitro *growth of some cancer cell lines, it could also stimulate the growth of normal cells [23]. Because there might be compounding effects due to the potential presence of catatonic agents at high bioactive concentrations mixed in with the desired bioactivity compounds, we further enriched the fractions.

Solvent partitioning based upon different solvent polarities was used for further purification and the antiproliferation active compounds were likely to be non-polar or low polar chemicals since the high cytotoxicity was principally observed in the hexane partitioned fraction (Figure 1). This agrees with Marcucci *et al*. who reported that the main chemical components in propolis were phenolic compounds of a low polarity [26]. Considering the physical appearance of the hexane-partitioned fraction, which was a light brown sticky liquid (Table 2), this contrasts to the crude ethanolic extract of the propolis as a rather dark solid. The change of extract characters after solvent partition based enrichment may represent the removal of some resin and wax from the original propolis. The difference in the observed IC_50 _(μg/ml) values for the antiproliferation activity between the five cancer cell lines (< 25) and the two normal cell lines (> 30) was evident (Figure 1), but not different enough to likely be clinically useful. Thus, the hexane-partitioned fraction was then further fractionated by quick column chromatography with elution based upon solvents of increasing polarity. In terms of the IC_50 _values for antiproliferation activity, the 30DCM fraction was the one of the most active fractions against the five cancer cell lines but uniquely showed in contrast a very poor activity against the two normal (control) cell lines, and thus showed a good selective antiproliferation activity. That it eluted from a 3:7 (v/v) ratio of CH_2_Cl_2_: hexane is still consistent with the notion that the active compounds are of low polarity. The physical appearance of the 30DCM fraction as a brown solid (Table 3) again supported the removal of other components including resin and wax. Regardless, the IC_50 _(μg/ml) value for the CH-liver control cell line was increased, whilst that for the five cancer cell lines was significantly decreased (Figure 2), although the variation in response between the cell lines was marked.

The further enrichment of fraction 30DCM by size exclusion chromatography yielded one fraction, 30DCM-F3, which provided the highest cytotoxicity on cancer cell lines but the lowest cytotoxicity on normal cells (Table 5).

**Figure 3:**
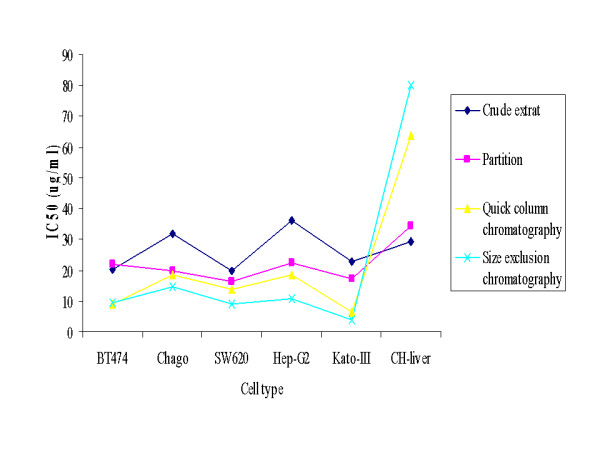
**The decrease in the *in vitro *IC_50 _values (μg/ml) on five cancer cell lines, plus the CH-liver and HS27 fibroblast normal cell lines as a comparative reference control, with increasing purification stages of the propolis extract from *T. laeviceps***. Data came from the mean ± 1 SD of percentage of cell viability which was derived from three replicates.

Furthermore, considering the TLC separation pattern, the more purification steps that were performed the better the observed separation and migration of compounds was (data not shown). Overall, the bioassay-guided isolation would appear to be suitable to use in order to obtain the purified active compounds (Figure 3), as reported before [18], but the other fractions need to be screened as well, and the fractions processed to pure compounds for confirmation. However, considering the results presented here, it is possible that the antiproliferation activity in each or some of the fractions is derived from a combination of compounds. Certainly, Orsolic *et al*. reported a synergistic antitumor effect by the water-soluble derivatives of propolis from *A. mellifera *in Croatia [27]. In the future, the fractional inhibitory concentration index (FICI) method should be performed in order to determine the level, if any, of synergy and antagonism. In addition, the purification to homogeneity and analysis of the chemical structures of each bioactive component should be performed in order to investigate which exact compounds in Thai propolis are responsible for the antiproliferation, and to act as the template for future drug design.

## Conclusions

Propolis of the stingless bee *(Trigona laeviceps) *from Thailand was tested for antiproliferative activity. Five cancer cell lines (Chago, Kato-III, SW620, BT474 and Hep-G2) and two normal cell lines (CH-liver cells and HS27 fibroblast cells) were selected for this purpose. The crude ethanolic extract displayed a good antiprolferative activity. For example, the IC_50 _was 19.9 and 29.1 μg/ml for the SW620 cancer cell line and CH-liver cells, respectively. After solvent partitioning, the hexane fractions revealed the highest antiproliferative activity against the five cancer cell lines and the lowest cytotoxic activity on the normal cell lines. For example, IC_50 _values of 16.4 and 32.4 μg/ml for the SW620 cancer cell line and CH-liver cells, respectively. The hexane fraction part was, therefore, purified in the next step by quick column and size exclusion chromatography. Considering the IC_50_, it was obvious that the more purified fractions were, the higher the antiproliferative activity was achieved. Two fractions that eluted with 30% and 100% (v/v) CH_2_Cl_2 _in hexane (30DCM and 100DCM, respectively) are potential sources of new antiproliferative compounds. Thus, *T. laeviceps *propolis from Thailand contains some bioactive compounds that are not only effective in antiproliferative activity on cancer cell lines, but also nontoxic to normal cell lines. In the future, it is possible that the bioactive compounds could serve as a template for anticancer-drug developing program.

## Competing interests

The authors declare that they have no competing interests.

## Authors' contributions

SU collected the samples, prepared the extracts and carried out the experiments. PP contributed to the study design, analyzed and interpreted the data. SP carried out some of the experiments. CC designed and supervised the experiments and contributed in drafting the manuscript. All authors read the manuscript, contributed in correcting it and approved its final version.

## Pre-publication history

The pre-publication history for this paper can be accessed here:

http://www.biomedcentral.com/1472-6882/11/37/prepub
